# Shape-Controlled TiO_2_ Nanomaterials-Based Hybrid Solid-State Electrolytes for Solar Energy Conversion with a Mesoporous Carbon Electrocatalyst

**DOI:** 10.3390/nano11040913

**Published:** 2021-04-03

**Authors:** Seung Man Lim, Juyoung Moon, Uoon Chul Baek, Jae Yeon Lee, Youngjin Chae, Jung Tae Park

**Affiliations:** 1 Department of Chemical Engineering, Konkuk University, 120 Neungdong-ro, Gwangjin-gu, Seoul 05029, Korea; smlim01km@gmail.com (S.M.L.); jymoon01kd@gmail.com (J.M.); ucbaek04km@gmail.com (U.C.B.); jylee05km@gmail.com (J.Y.L.); 2 Faculty of Industrial Design Engineering, Delft University of Technology, Landbergstraat 15, 2628 CE Delft, The Netherlands; mariechae@gmail.com

**Keywords:** solid-state electrolyte, titanium dioxide (TiO_2_), one-dimensional (1D), photoelectrochemical, mesoporous carbon, dye-sensitized solar cell (DSSC)

## Abstract

One-dimensional (1D) titanium dioxide (TiO_2)_ is prepared by hydrothermal method and incorporated as nanofiller into a hybrid polymer matrix of polyethylene glycol (PEG) and employed as a solid-electrolyte in dye-sensitized solar cells (DSSCs). Mesoporous carbon electrocatalyst with a high surface area is obtained by the carbonization of the PVDC-*g*-POEM double comb copolymer. The 1D TiO_2_ nanofiller is found to increase the photoelectrochemical performance. As a result, for the mesoporous carbon-based DSSCs, 1D TiO_2_ hybrid solid-state electrolyte yielded the highest efficiencies, with 6.1% under 1 sun illumination, in comparison with the efficiencies of 3.9% for quasi solid-state electrolyte and 4.8% for commercial TiO_2_ hybrid solid-state electrolyte, respectively. The excellent photovoltaic performance is attributed to the improved ion diffusion, scattering effect, effective path for redox couple transfer, and sufficient penetration of 1D TiO_2_ hybrid solid-state electrolyte into the electrode, which results in improved light-harvesting, enhanced electron transport, decreased charge recombination, and decreased resistance at the electrode/electrolyte interface.

## 1. Introduction

Dye-sensitized solar cells (DSSCs) composed of mesoporous metal oxide-based photoanode, the precious-metal-based counter electrode, and liquid state electrolyte have attracted, over the last decade, considerable interest because of attractive characteristics such as high photoelectrochemical performance, low production cost, and ease of processing [1,2]. However, the liquid electrolyte-based DSSC has some drawbacks. Due to the leakage and volatilization of solvent, the photoelectrochemical performance of liquid electrolyte-based DSSC decreases with time, thereby suggesting that the electrolyte plays a key factor in improving the stability of DSSCs. An ideal electrolyte in DSSCs should have several advantages, such as thermal and chemical stability, a low absorption coefficient in the visible region, less leakage and volatilization, and high ion conductivity and diffusivity [3,4,5,6].

Several researchers have reported the use of poly(ethylene oxide) (PEO), poly(ethylene glycol) (PEG), and poly(methyl methacrylate) (PMMA) in DSSCs as quasi solid-state electrolytes [7,8,9,10,11,12,13]. The utilization of quasi solid-state electrolytes has many advantages, such as thermal and chemical stability and less leakage and evaporation of solvent. However, the photoelectrochemical performance of quasi solid-state electrolyte is lower than that of liquid state-electrolyte due to low ion diffusivity and poor permeability into mesoporous metal oxide based photoanode. Therefore, developing a quasi-solid-state or solid-state electrolyte with high conductivity and good interfacial contact with photoanode and counterelectrode is challenging.

The introduction of shape-controlled nanomaterials as nanofillers into the solid-state electrolyte is a simple and facile suggestion for improving photoelectrochemical performances, which reduces the crystallinity of the polymer main chain in the electrolyte and improves the ionic conductivity due to increased free volume for redox couple transport. In general, metal oxides such as WO_3_, SiO_2_, SnO_2_, and titanium dioxide (TiO_2)_, which are cost-effective and offer easy control of morphology, are utilized as nanofillers [14,15,16,17,18,19,20,21,22]. However, a quantitative comparison between one-dimensional (1D) TiO_2_ and commercial TiO_2_-based solid electrolytes in photoelectrochemical applications has not been conducted often because it is difficult to control the shape and size of nanomaterials.

Meanwhile, earth abundant mesoporous carbon materials present a great potential replacement of precious-metal-based counter electrode because of their high electrocatalytic activity, good electrochemical stability, and large surface area for rapid interface reactions [23,24]. In this regard, it is reported to be possible to directly prepare mesoporous carbon nanostructure by self-assembling a block copolymer as carbon precursors [25]. However, double comb copolymer-derived mesoporous carbon counterelectrodes for DSSCs based on quasi solid-state or solid-state electrolytes are still in their infancy. Therefore, a systematic study is needed to clarify how a mesoporous carbon electrocatalyst and the shape-controlled nanomaterials based solid-state electrolyte impact on electrochemical and optical properties and their impact on the performance of photovoltaic devices.

In this study, the effect of the shape-controlled TiO_2_ nanomaterials in the hybrid solid-state electrolytes on the photoelectrochemical characteristics of mesoporous carbon-based DSSCs was investigated systematically. A mesoporous carbon electrocatalyst with a high surface area is obtained by the carbonization of the PVDC-*g*-POEM double comb copolymer. A nanofiller material in the form of 1D TiO_2_ was added to the hybrid solid-state electrolyte to enhance the ion diffusion, scattering effect, redox couple transfer, and penetration of electrolyte into the electrode, therefore improving the photochemical performance of the mesoporous carbon-based DSSCs. This research provides an essential first step toward the use of hybrid solid-state electrolytes containing shape-controlled TiO_2_ nanomaterials in mesoporous carbon-based photoelectrochemical applications.

## 2. Experimental Section

### 2.1. Materials

In this study, the chemicals were used as obtained, i.e., no further purification was conducted. Titanium (IV) butoxide (TTBT, 97%), HCl (37 wt%), I_2_, LiI, titanium diisopropoxide bis(ethylacetoacetate), 1-methyl-3-propylimidazolium iodide (MPII), PEG (M_w_ = 10,000 g mol^−1^), and H_2_PtCl_6_ were purchased from Sigma-Aldrich (St. Louis, MO, USA). Acetonitrile, 2-propanol, and chloroform were purchased from J. T. Baker (Thermo Fisher Scientific, Waltham, MA, USA). Deionized water (>18 MΩm) was obtained with a water purification system made by MilliporeSigma (Burlington, MA, USA). Commercial TiO_2_ paste (Ti-Nanoxide, D20) and ruthenium dye (N719) were purchased from Solaronix SA (Aubonne, Switzerland). Commercial TiO_2_ powder (P25) was prepared by Evonik Industries AG (formerly Degussa, Essen, Germany). Fluorine-doped tin oxide (FTO, TEC-7) conducting substrate was purchased from Pilkington, France.

### 2.2. Preparation of 1D TiO_2_ Hybrid Solid-State Electrolyte

The one-dimensional TiO_2_ nanorod (1D TiO_2_) deposited onto FTO substrate was synthesized via hydrothermal method [26]. First, 0.8 mL of titanium (IV) butoxide (TTBT) was added into 60 mL of 6 M HCl solution. FTO substrate (3 × 4 cm) was cleaned with chloroform and 2-propanol and placed in a Teflon-lined autoclave, wherein the conductive side of the FTO was placed face down. Then, the TTBT/HCl solution was transferred into the Teflon-lined autoclave followed by heating at 150 °C for 6 h. After reaction, 1D TiO_2_ deposited-FTO substrate was cleaned with water and ethanol several times and dried at 50 °C for 30 min. Then, 1D TiO_2_ were physically scrapped off from the 1D TiO_2_ deposited-FTO substrate with a slide glass. 1D TiO_2_ hybrid solid-state electrolyte were prepared with 1g of PEG, 0.15 g of LiI, 0.15g of 0.03 g of MPII, and 10 mL of I2 in acetonitrile with the addition of the 0.09 g of 1D TiO_2_ prepared in this study. It should be noted that 1D TiO_2_ nanomaterials in the solid-state electrolyte were well dispersed due to the plentiful amount of hydroxyl group of metal oxides. In addition, quasi solid-state electrolytes were prepared according to a previous report as control samples [27]. And commercial TiO_2_ hybrid solid-state electrolytes were prepared from PEG-based electrolyte with commercial TiO_2_ as a control.

### 2.3. Preparation of Mesoporous Carbon Electrocatalyst Based Counterelectrode

The mesoporous carbon electrocatalyst-based counter electrode was prepared by depositing on the prepared FTO substrate according to the modified protocol previously reported by our group [28]. In brief, 0.05 g of PVDC-*g*-POEM double comb copolymer was first dissolved in 1.5 mL of THF. Separately, 0.15 mL of concentrated HCl (37 wt%) was slowly added to 0.15 mL of deionized water at room temperature under stirring speed of 500 rpm. After aging for 15 min at room temperature, the HCl/H_2_O solution was slowly added to the PVDC-*g*-POEM double comb copolymer solution with a stirring speed of 300 rpm, followed by annealing at 60 °C for 1h to obtain a viscous solution. Then, the viscous PVDC-*g*-POEM double comb copolymer solution was screen-printed on the FTO substrate, and the printed film was calcinated at 550 °C in Ar for 30 min.

### 2.4. Preparation of 1D TiO_2_ Hybrid Solid-State Electrolyte Based DSSCs

The FTO substrate was cleaned with chloroform and 2-propanol. As a photoanode, the cleaned FTO conductive side was spin coated with 1 wt% titanium diisopropoxide bis(ethylacetoacetate) 2-butanol solution followed by calcination at 450 °C for 30 min. To prepare nanocrystalline TiO_2_, commercial TiO_2_ paste (Ti-Nanoxide, D20) was deposited on the prepared FTO substrate using the doctor-blade method and dried at 50 and 80 °C for 1 h, respectively, followed by sintering at 450 °C for 30 min. Then, the prepared photoanode was immersed in 0.1 mM N719 dye ethanol solution at 50 °C for 3 h. According to a previously reported procedure, DSSCs with an active area of 0.4 cm^2^ were constructed by drop-casting of 1D TiO_2_ hybrid solid-state electrolyte solution onto the photoanode and covering with the counterelectrode [29]. And then, the DSSCs were dried in a drying oven at 50 °C for 24 h for solvent evaporation.

### 2.5. Characterization

The surface and cross-sectional morphologies were acquired by field emission scanning electron microscopy (FE-SEM) (SU8010, Hitachi, Tokyo, Japan). Transmission electron microscope (TEM) images were obtained from a Philips CM30 microscope operating at 300 kV to observe nanostructures of samples (Philips CM30, Amsterdam, The Netherlands). The crystalline structure of 1D TiO_2_ was obtained by X-ray diffraction (XRD) spectroscopy (Smartlab, Rigaku, Tokyo, Japan) with CuKα radiation (λ = 1.5406 Å). The specific surface area and pore volume of the mesoporous carbon were measured using the N2 adsorption-desorption isotherm via the Brunauer-Emmett-Teller (BET) methods. Before the adsorption-desorption measurement, the mesoporous carbon was degassed at 70 °C under a dynamic vacuum (10^−2^ Torr) for 1 h. Intensity-modulated photocurrent/voltage spectroscopy (IMPS/IMVS) was used to investigate electron diffusion and recombination properties. UV-vis diffuse-reflectance spectra were characterized by spectrophotometry (Mega-900, Scinco, Seoul, Korea). Thickness of photoanode and counterelectrode was measured using an alpha-step IQ surface profile (KLA Tencor). The current density–voltage (*J–V*) measurements were performed using a potentiostat (Compactstat.h, Ivium Technologies, Eindhoven, The Netherlands). The solar simulator equipped with a 1000 W xenon lamp was used as light source (SunLite, Abet Technologies, Inc., Milford, CT, USA). Electrochemical impedance spectroscopy (EIS) characteristics were measured by a potentiostat under open-circuit voltage in a frequency range of 0.01–100 kHz. Incident photo-to-electron conversion efficiency (IPCE) spectra were obtained by IPCE system (IPCEPEIPCE100S, HS Technologies, Plano, TX, USA). The photovoltaic parameters were calculated using Equations (1) and (2):

*FF* = *V_max_* × *J_max_*/*V_oc_* × *J_sc_* (1)

*η* = *V_max_* × *J_max_/P_in_* × 100 = *V_oc_* × *J_sc_* × *FF/P_in_* × 100 (2)

where *η* is the photovoltaic conversion efficiency, *J_sc_* is short-circuit current density, *V_oc_* is the open-circuit voltage, *P_in_* is incident light power, *FF* is fill factor, and *J_max_* and *V_max_* are current density and voltage, respectively, when maximum power is achieved.

## 3. Results and Discussion

**Scheme 1:**
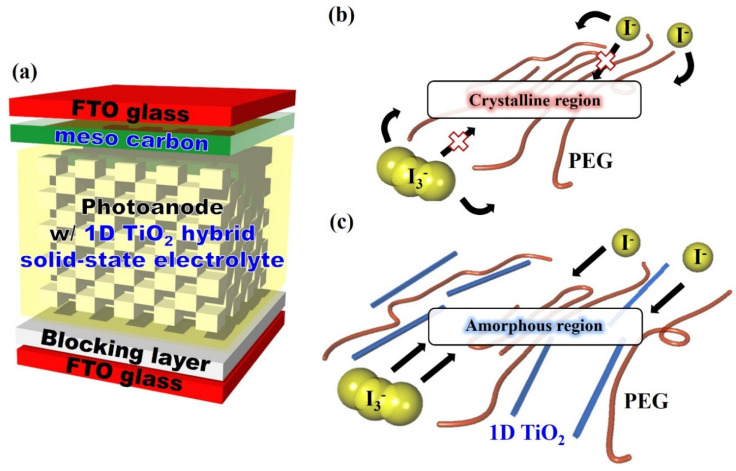
(**a**) Schematic of the dye-sensitized solar cells (DSSCs) fabricated in this study and the possible redox couple reaction of (**b**) quasi solid-state electrolyte and (**c**) One-dimensional (1D) titanium dioxide (TiO_2)_ hybrid solid-state electrolyte.

The schematic of the dye-sensitized solar cells (DSSCs) fabricated in this study and the possible redox couple reaction in two types of electrolytes are shown in Scheme 1. In quasi-solid-state electrolytes, the redox couple transfers in several ways, such as polymer segment motion or ion diffusion. However, redox couple transfer is restricted by high crystallinity of the PEG crystalline region of quasi-solid-state electrolyte, resulting in low ionic conductivity. Therefore, reducing the crystallinity of the polymer main chain is a key factor for improving the photoelectrochemical performance of DSSCs [30,31]. The hydrophilic 1D TiO_2_ interacted with the PEG main chain in the 1D TiO_2_ hybrid solid-state electrolyte, resulting in an amorphous region, which created free volume from the crystalline region. This amorphous region could be an effective path for redox couple transfer, thereby resulting in enhanced ionic conductivity.

**Figure 1:**
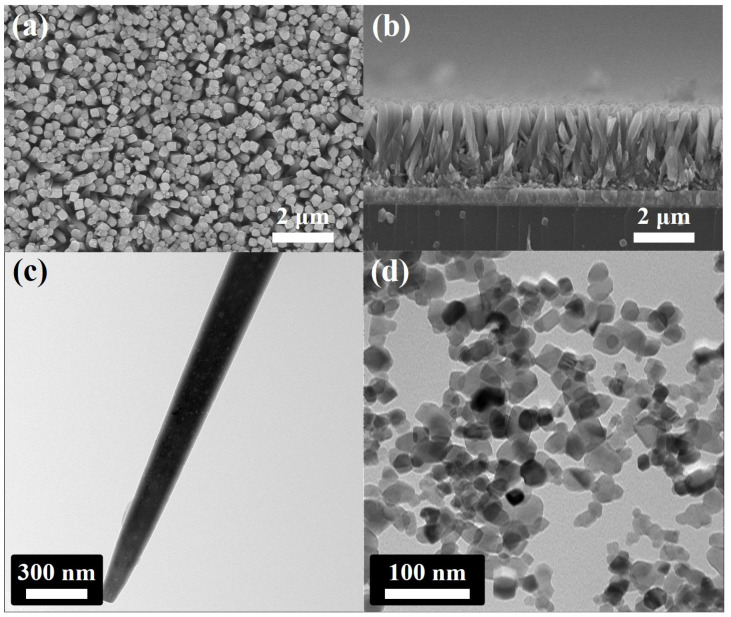
Field emission scanning electron microscopy (FE-SEM) images of vertically aligned 1D TiO_2_ grown on Fluorine-doped tin oxide (FTO) glass via the hydrothermal process: (**a**) top-view and (**b**) cross-section of 1D TiO_2_. Transmission electron microscope (TEM) image of (**c**) 1D TiO_2_ and (**d**) commercial TiO_2_.

The SEM and TEM images of 1D TiO_2_ and commercial TiO_2_ are shown in Figure 1. The surface of the FTO was homogeneously covered with vertically aligned 1D TiO_2_ with lengths of 3 μm and diameters of 250 nm, as shown in Figure 1a,b. It should be noted that the crystalline phase of the FTO substrate was similar to that of 1D TiO_2_; the 1D TiO_2_ nanostructure was grown on FTO without the Ti seed layer [32]. Figure 1c shows the TEM image of a 1D TiO_2_ with a diameter of about 250 nm, which is consistent with the SEM images. Moreover, the commercial TiO_2_ agglomerated to some degree with 30 nm in size, as shown in Figure 1d. Note that Figure S1 shows the size distribution of commercial TiO_2_ and 1D TiO_2_. The average diameter of the commercial TiO_2_ was approximately 20~30 nm. The diameter of the 1D TiO_2_ was approximately 240~250 nm.

**Figure 2:**
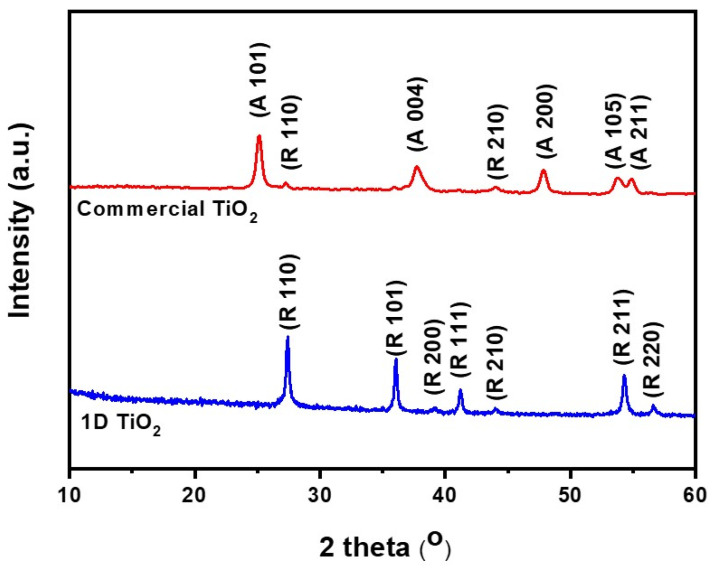
X-ray diffraction (XRD) patterns of 1D TiO_2_ and commercial TiO_2_ where R and A denote rutile and anatase phases, respectively.

Figure 2 shows the XRD pattern of the as-prepared 1D TiO_2_ and commercial TiO_2_. 1D TiO_2_ showed several sharp peaks at 2 thata values of 27.5°, 36.2°, 38.4°, 41.4°, 44.4°, 54.5°, and 56.6°, corresponding to (110), (101), (200), (111), (210), (211), and (220) crystal planes of the rutile TiO_2_ phase, respectively (ICDD-JCPDS database, no. 77-0441). The commercial TiO_2_ exhibited several sharp peaks centered at 2 theta values of 25.5°, 38.0°, 48.2°, 54.3°, and 55.2°, which correspond to the (101), (004), (200), (105), and (211) reflections, respectively, of the anatase form of TiO2 (JCPDS no. 21-1272). These results indicate the successful preparation of highly crystalline 1D TiO_2_ from the TiO_2_ metal oxide precursor using the hydrothermal process. According to the Mie theory, the superior scattering center is found in those with sizes comparable to the wavelength of light, and the light scattering occurs due to higher incidence of light trapping in photovoltaic devices [33]. Therefore, we can expect that high-aspect-ratio materials such as 1D TiO_2_ in rutile phase give rise to an improved light scattering effect compared to low-aspect-ratio materials such as commercial TiO_2_ in anatase phase, leading to enhanced photovoltaic performance. Moreover, replacing our suggested rutile phase of 1D TiO_2_ with commercial rutile phase TiO_2_ as a nanofiller is expected to vary the photoelectrochemical characteristics, which are currently investigated in our lab.

**Figure 3:**
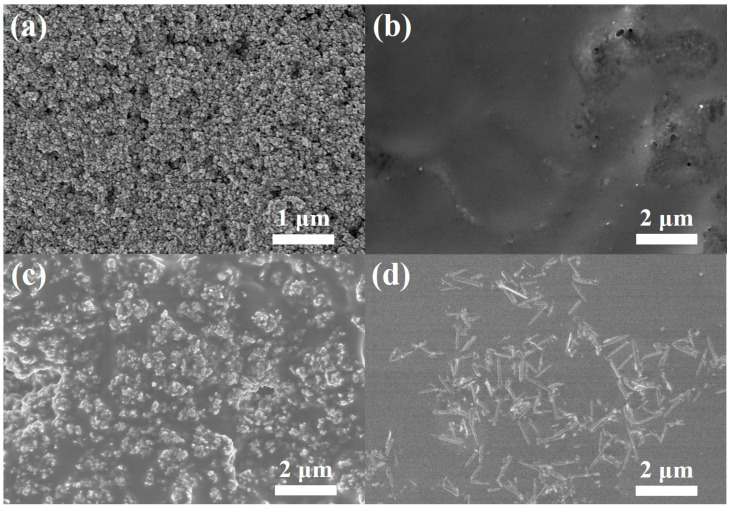
SEM image of the (**a**) photoanode, (**b**) photoanode after quasi solid-state electrolyte casting, (**c**) photoanode after commercial TiO_2_ hybrid solid-state electrolyte casting and (**d**) photoanode after 1D TiO_2_ hybrid solid-state electrolyte casting.

Interfacial contact between the photoanode and the electrolyte is of importance in determining the photoelectrochemical performance of DSSCs. Therefore, the top-view SEM images of the photoanode were taken before and after casting (quasi) solid-state electrolytes, as shown in Figure 3. Before electrolyte casting, the metal oxide nanoparticles had a size of 30 nm in photoanode, and the pores between the structures were detectable, as shown in Figure 3a. Figure 3b presents a SEM image for a top-view of a quasi-solid-state electrolyte filled mesoporous photoanode. The quasi-solid-state electrolyte uniformly covered the mesoporous photoanode without any noticeable defective sites. Figure 3c,d presents an SEM image for a top-view of the mesoporous photoanode with commercial TiO_2_ hybrid solid-state electrolyte and 1D TiO_2_ hybrid solid-state electrolyte, respectively. The commercial TiO_2_ maintains the morphological structure in the polymer matrix, showing a nanoscale range. Moreover, the 1D TiO_2_ in the 1D TiO_2_ hybrid solid-state electrolyte indicates that the 1D TiO_2_ plays a role as an effective light scattering center. It should be noted that commercial TiO_2_ or 1D TiO_2_ nanomaterials lead to the enhanced interaction between electrolyte and photoanode, resulting in PEG-based electrolyte remaining in solids and behaving as highly viscous liquids. In addition, the thickness of photoanodes was increased from 7 to 10 μm after commercial TiO_2_ and 1D TiO_2_ hybrid solid-state electrolyte casting.

**Figure 4:**
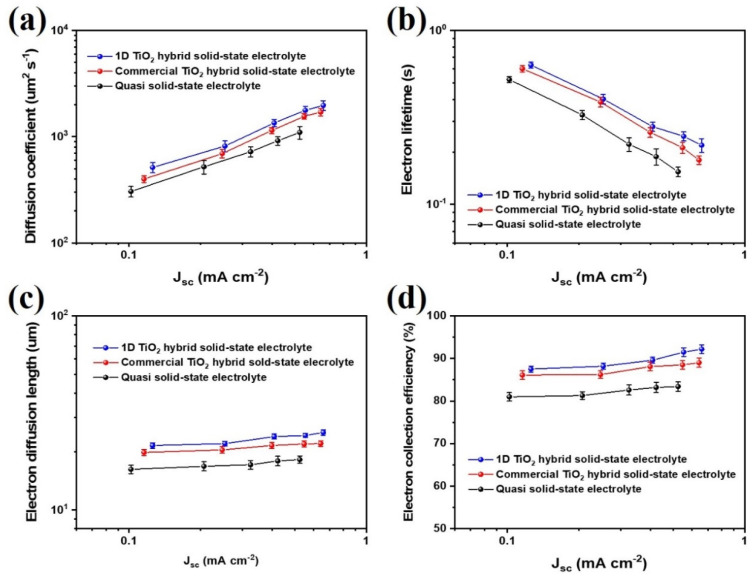
(**a**) Diffusion coefficients (*D_n_*), (**b**) recombination time (*τ_r_*), (**c**) electron diffusion length (*L_n_*), and (**d**) electron collection efficiency (*η_cc_*) as functions of the *J_sc_* for the mesoporous carbon-based dye-sensitized solar cells (DSSCs) with quasi solid-state electrolyte, commercial TiO_2_ hybrid solid-state electrolyte and 1D TiO_2_ hybrid solid-state electrolyte measured by IMPS and IMVS. Error bars represent the standard deviation of at least 5 cells.

The electron transport properties of 1D TiO_2_ hybrid solid-state electrolyte, including diffusion coefficient (*D_n_*), electron lifetime (*τ_r_*), electron diffusion length (*L_n_*), and electron collection efficiency (*η_cc_*) as a function of *J_sc_* were characterized using IMPS/IMVS analysis, as shown in Figure 4. IMPS/IMVS analyses are useful for obtaining electron transport time (*τ_t_*) and electron lifetime (*τ_r_*). Then, the electron transport time was converted to diffusion coefficient (*D_n_*) as follows:

*D_n_* = d^2^/2.35 *τ_t_* (3)

where *d* is the film thickness of photoanode. In addition, electron diffusion length (*L_n_*), and electron collection efficiency (*η_cc_*) were obtained as follows:

*L_n_* = (*D_n_τ_r_*)^0.5^ (4)

*η_cc_* = 1 − *τ_t_/τ_r_* (5)

where *τ_r_* and *τ_t_* values are obtained using IMVS and IMPS analysis, respectively [34,35]. The *D_n_* and *τ_r_* values of DSSCs based on 1D TiO_2_ hybrid solid-state electrolyte were higher than that of quasi solid-state electrolyte and commercial TiO_2_ hybrid solid-state electrolyte, indicating that the introduction of 1D TiO_2_ as nanofiller in electrolyte is a good suggestion to enhance electron transfer of photoelectrochemical devices. The *L_n_* and *η_cc_* values of the 1D TiO_2_ hybrid solid-state electrolyte are higher than those of the quasi solid-state electrolyte for the entire range of *Jsc*. This demonstrates that it is the amorphous region of 1D TiO_2_ hybrid solid-state electrolyte which is responsible for enhanced charge transport and reduced recombination in the photoelectrochemical devices. It should be noted that the 1D TiO_2_ hybrid solid-state electrolyte provides a more effective path for redox couple transfer than commercial TiO_2_ hybrid solid-state electrolyte due to the facile formation of an amorphous region.

**Figure 5:**
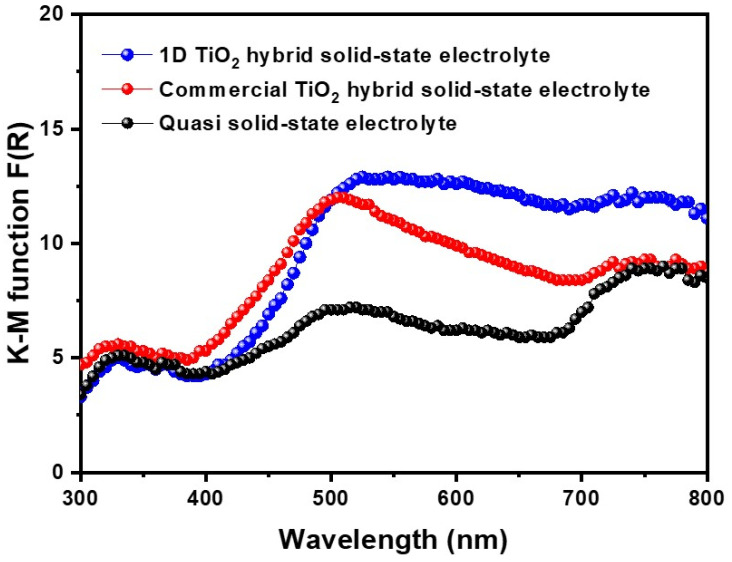
Kubelka-Munk (KM) function of quasi solid-state electrolyte, commercial TiO_2_ hybrid solid-state electrolyte, and 1D TiO_2_ hybrid solid-state electrolyte.

To investigate the scattering effect of 1D TiO_2_ hybrid solid-state electrolyte, diffuse reflectance of DSSCs fabricated with symmetric photoanodes was characterized, as shown in Figure 5. In the short wavelength region, i.e., 300~450 nm, there was no difference in the diffuse reflectance between the quasi solid-state electrolyte and two types of hybrid solid-state electrolytes. However, in the wavelength region of 450–600 nm, the 1D TiO_2_ hybrid solid-state electrolyte had higher diffuse reflectance than the quasi solid-state electrolyte and commercial TiO_2_ hybrid solid-state electrolyte, suggesting that the use of 1D TiO_2_ induced scattering effect, which resulted in higher light-harvesting properties. It should be noted that the adding of a high-aspect-ratio nanomaterial is an effective method for enhancing the light scattering capacity of photovoltaic devices, which increases an equivalent optical path and thereby enhances a photocurrent density [14]. Moreover, replacing our suggested 1D TiO_2_ with different aspect ratio nanomaterials or large particles as a nanofiller is expected to improve the light scattering property, which is currently being investigated in our lab.

**Figure 6:**
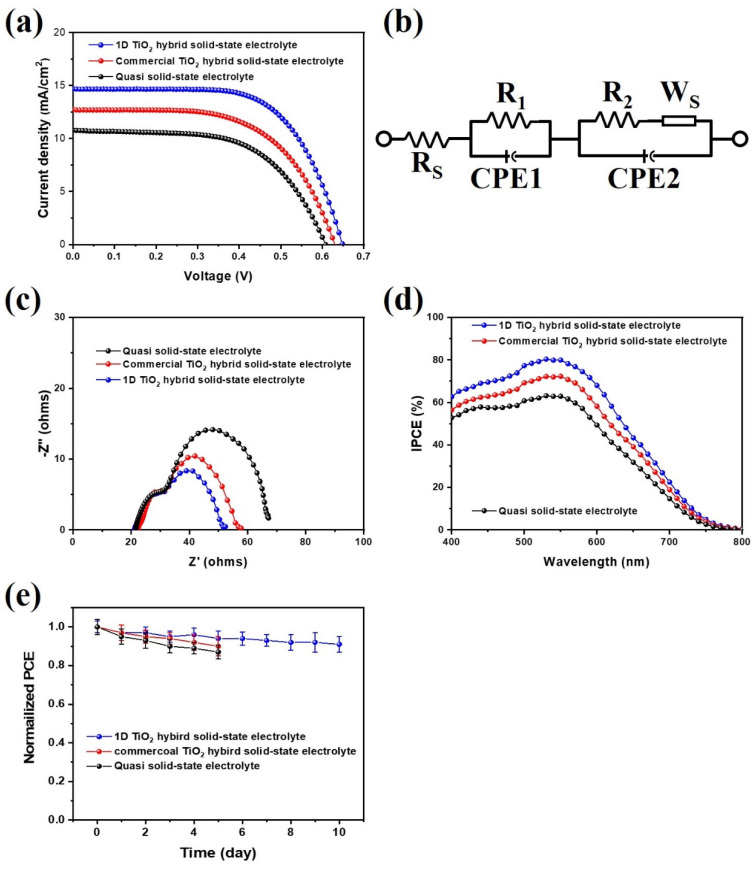
(**a**) *J-V* curves, (**b**) equivalent circuit diagram, (**c**) Electrochemical impedance spectroscopy (EIS) Nyquist plots, and (**d**) Incident photo-to-electron conversion efficiency (IPCE) curves of mesoporous carbon-based DSSCs fabricated with quasi solid-state electrolyte, commercial TiO_2_ hybrid solid-state electrolyte, and 1D TiO_2_ hybrid solid-state electrolyte at 100 mW/cm^2^. (**e**) Normalized cell efficiency of the mesoporous carbon-based DSSCs with 1D TiO_2_ hybrid solid-state electrolyte (40–50% relative humidity). Error bars represent the standard deviation of at least 5 cells.

To investigate the photovoltaic properties of mesoporous carbon-based DSSCs with 1D TiO_2_ hybrid solid-state electrolytes, the current density-voltage (*J-V*) measurement curves are shown in Figure 6a. The photovoltaic parameters, short-circuit current density (*Jsc*), open-circuit voltage (*Voc*), *FF*, and overall energy conversion efficiency (*η*) are listed in Table 1. It should be noted that the mesoporous carbon derived from PVDC-*g*-POEM double comb copolymer showed the BET surface area of 472.15 m^2^/g and highly ordered porosity, as results in one of the best counterelectrode materials for photoelectrochemical devices. (Figure S2) [28] For reliability, we additionally provide photovoltaic parameters of the different sets of mesoporous carbon-based DSSCs with 1D TiO_2_ hybrid solid-state electrolytes. The photovoltaic parameters with error bars of the standard deviations for at least five cells were summarized in Figure S3 and Table S1. For a mesoporous carbon-based DSSCs, using a quasi-solid-state electrolyte, *Jsc* and *Voc* are 10.7 mA cm^−2^ and 0.60 V, respectively, whereas the *FF* and the *η* are 0.59 and 3.9%, respectively. In comparison, the solar conversion efficiency of the 1D TiO_2_ hybrid solid-state electrolyte was the highest achieved in this research (6.1%); this correlates with the highest *Jsc* of 14.6 mA cm^−2^, a *Voc* of 0.65 V, and a *FF* of 0.64. It should be noted that the efficiency of the mesoporous carbon-based DSSCs using the 1D TiO_2_ hybrid solid-state electrolyte represents one of the highest values reported for Pt-free counterelectrode-based DSSCs to date, as shown in Table S2 [36,37,38,39,40]. Moreover, the carbon-based DSSCs fabricated with commercial TiO_2_ hybrid solid-state electrolyte show efficiency of 4.8% with a *Jsc*, *Voc,* and *FF* of 12.7 mA cm^−2^, 0.63 V, and 0.60, respectively. The mesoporous carbon-based DSSCs with 1D TiO_2_ hybrid solid-state electrolyte achieved more than 30% *Jsc* value compared to the quasi solid-state electrolyte, demonstrating that the introduction of 1D TiO_2_ to electrolyte enhanced ion diffusion of the redox couple as well as the scattering effect, thereby improving the light-harvesting. In addition, a slightly higher *Voc* value was observed in mesoporous carbon-based DSSCs with 1D TiO_2_ hybrid solid-state electrolyte. Moreover, the *Voc* value can be obtained as follows:

*Voc* = |*V_fb_* − *V_red_*| (6)

where *V_fb_* is the Fermi level potential of photoanode and *V_red_* is the reduction potential of redox couple in electrolyte [41]. Possible contributions to the enhanced *Voc* value include the decreased charge recombination, electron back reaction loss, and the reduction of the *V_red_* of the electrolyte by the 1D TiO_2_. The decreasing of the charge recombination and electron back reaction loss in mesoporous carbon-based DSSCs with 1D TiO_2_ hybrid solid-state electrolyte were also characterized using previous IMPS/IMVS measurements. The Nyquist plots and equivalent circuit diagram of the mesoporous carbon-based DSSCs with 1D TiO_2_ hybrid solid-state electrolyte are shown in Figure 6b,c, respectively. The electrochemical parameters of mesoporous carbon-based DSSCs are listed in Table 2. An equivalent circuit model consisted of the series resistance (*R_s_*), which is ohmic resistance of the FTO substrate, counterelectrode/electrolyte interfacial charge transfer resistance (*R_1_*), photoanode/electrolyte interfacial charge transfer resistance (*R_2_*), and Warburg diffusion resistance (*W_s_*). Upon analysis of Nyquist impedance data through fitting with the equivalent circuit, the total resistance values of mesoporous carbon-based DSSCs with 1D TiO_2_ hybrid solid-state electrolyte were significantly reduced compared to the quasi solid-state electrolyte. This phenomenon may be explained by the increased electron transport in mesoporous carbon-based DSSCs with 1D TiO_2_ hybrid solid-state electrolyte, leading to decreased resistance at the photoanode/electrolyte interface. Incident photon-to-electron conversion efficiency (IPCE) quantifies the internal quantum and light-harvesting efficiencies at an incident wavelength for DSSCs. Figure 6d presents the IPCE spectra for mesoporous carbon-based DSSCs with the 1D TiO_2_ hybrid solid-state electrolyte. The DSSCs based on the 1D TiO_2_ hybrid solid-state electrolyte showed the maximum IPCE of about 80%, while the DSSCs based on the quasi solid-state electrolyte exhibited the maximum IPCE of about 60%. Furthermore, the *Jsc* values from the IPCE curves were quite consistent with those determined from *J-V* curves for all of the mesoporous carbon-based DSSCs, as shown in Table 1. The enhanced IPCE value of the mesoporous carbon-based DSSCs with the 1D TiO_2_ hybrid solid-state electrolyte was attributed to enhanced light harvesting efficiency, consistent with the previous diffuse reflectance. Furthermore, Figure 6e showed the stability behaviors of the mesoporous carbon-based DSSCs with the 1D TiO_2_ hybrid solid-state electrolyte. After 10 days, the cell efficiency of its devices remained stable, suggesting that the mesoporous carbon and 1D TiO_2_ hybrid solid-state electrolyte have good electrochemical stability and enhanced electrode/electrolyte interface. Moreover, it should be noted that the cell efficiency of quasi solid-state electrolyte and commercial TiO_2_ hybrid solid-state electrolyte is lower than that of 1D TiO_2_ hybrid solid-state electrolyte after stability testing.

## 4. Conclusions

1D TiO_2_ was prepared by hydrothermal method and used as a nanofiller in a PEG-based hybrid solid-state electrolyte to enhance the ion diffusion, scattering effect, effective path for redox couple transfer, and sufficient penetration of hybrid solid-state electrolyte into the electrode, thereby improving the photoelectrochemical performance of mesoporous carbon-based DSSCs. Mesoporous carbon electrocatalyst was prepared using a carbonization approach with PVDC-*g*-POEM double comb copolymers. The mesoporous carbon-based DSSCs based on 1D TiO_2_ hybrid solid-state electrolyte (6.1%) showed a higher cell performance than those with quasi solid-state electrolyte (4.8%) and commercial TiO_2_ hybrid solid-state electrolyte (3.9%). These results indicate that the addition of a 1D TiO_2_ nanofiller to a hybrid solid-state electrolyte improves the light-harvesting and charge transport, reduces recombination, and decreases the resistance at the electrode/electrolyte interface. We believe this suggestion presents a promising way to prepare hybrid solid-state electrolytes with enhanced performance for mesoporous carbon-based photoelectrochemical applications.

## Figures and Tables

**Table 1:** Photovoltaic properties of mesoporous carbon-based dye-sensitized solar cells (DSSCs) with quasi solid-state electrolyte, commercial TiO_2_ hybrid solid-state electrolyte, and 1D TiO_2_ hybrid solid-state electrolyte at 100 mW/cm^2^ (AM 1.5) ^a, b, c, d, e, f, g, h, i^.

Electrolyte	*Voc* (V)	*Jsc ^g^* (mA/cm^2^)	*Jsc ^h^* (mA/cm^2^)	*FF*	*η* (%)
Quasi solid-state electrolyte	0.60 ± 0.04	10.7 ± 0.7	10.3	0.59 ± 0.02	3.9 ± 0.20
Commercial TiO_2_ hybrid solid-state electrolyte	0.63 ± 0.04	12.7 ± 0.4	12.0	0.60 ± 0.01	4.8 ± 0.15
1D TiO_2_ hybrid solid-state electrolyte	0.65 ± 0.03	14.6 ± 0.6	13.8	0.64 ± 0.03	6.1 ± 0.20

^a^ Quasi solid-state electrolyte consists of PEG, LiI, MPII, and I_2_ in acetonitrile. ^b^ Commercial TiO_2_ hybrid solid-state electrolyte consist of commercial TiO_2_, PEG, LiI, MPII, and I_2_ in acetonitrile. ^c^ 1D TiO_2_ hybrid solid-state electrolyte consist of 1D TiO_2_, PEG, LiI, MPII, and I_2_ in acetonitrile. ^d^ The photoactive area for DSSCs was 0.16 cm^2^. ^e^ The thickness of the photoanode was approximately 7 μm. ^f^ The thickness of the counterelectrode was approximately 5 μm. ^g^ Determined from *J-V* curves. ^h^ Determined from IPCE curves. ^i^ Error bars represent the standard deviation of at least 5 cells.

**Table 2:** Electrochemical parameters of mesoporous carbon-based DSSCs with quasi solid-state electrolyte, commercial TiO_2_ hybrid solid-state electrolyte and 1D TiO_2_ hybrid solid-state electrolyte, as determined by EIS analysis at 100 mW/cm^2^ (AM 1.5). Error bars represent the standard deviation of at least 5 cells.

Electrolyte	R_s_ (Ω)	R_1_ (Ω)	R_2_ (Ω)	W_s_ (Ω)
Quasi solid-state electrolyte	21.6 ± 3.5	9.8 ± 1.5	34.4 ± 5.0	2.4 ± 0.3
Commercial TiO_2_ Hybrid solid-state electrolyte	22.3 ± 3.0	8.7 ± 1.2	25.5 ± 4.5	2.0 ± 0.3
1D TiO_2_ Hybrid solid-state electrolyte	21.1 ± 3.5	9.6 ± 1.5	20.1 ± 4.0	1.8 ± 0.2

## Data Availability

No data available.

## Supplementary Materials

The following are available online at https://www.mdpi.com/article/10.3390/nano11040913/s1, Figure S1: The size distribution of commercial TiO_2_ (a) and 1D TiO_2_ (b). Figure S2: (**a**) N_2_ adsorption (filled symbols) and desorption (unfilled symbols) isotherms of mesoporous carbon electrocatalyst derived from PVDC-*g*-POEM double comb copolymer and (**b**) SEM of mesoporous carbon electrocatalyst. Figure S3: Photovoltaic parameters of mesoporous carbon-based DSSCs fabricated with quasi solid-state electrolyte, commercial TiO_2_ hybrid solid-state electrolyte and 1D TiO_2_ hybrid solid-state electrolyte at 100 mW/cm^2^. Error bars represent the standard deviation of at least 5 cells. Table S1: Photovoltaic parameters of mesoporous carbon-based dye-sensitized solar cells (DSSCs) with quasi solid-state electrolyte, commercial TiO_2_ hybrid solid-state electrolyte, and 1D TiO_2_ hybrid solid-state electrolyte at 100 mW/cm^2^ (AM 1.5). Table S2: Comparison of photovoltaic parameters of Pt-free counterelectrode based DSSCs fabricated with solid-state electrolytes reported in the literature.
