# Supplementary material for: Shape-Controlled TiO2 Nanomaterials-Based Hybrid Solid-State Electrolytes for Solar Energy Conversion with a Mesoporous Carbon Electrocatalyst
Source: Nanomaterials (Basel). 2021 Apr 3;11(4):913. doi: 10.3390/nano11040913 (PMC8066460; doi:10.3390/nano11040913)
Supplement: Supplementary file 1 [file nanomaterials-11-00913-s001.pdf]

Supplementary Information

Shape-Controlled TiO_2 Nanomaterials-Based Hybrid Solid-State Electrolytes for Solar Energy Conversion with a Mesoporous Carbon Electrocatalyst

Seung Man Lim [1], Juyoung Moon [1], Uoon Chul Baek [1], Jae Yeon Lee [1], Youngjin Chae [2] and Jung Tae Park [1,*]

[1] Department of Chemical Engineering, Konkuk University, 120 Neungdong-ro, Gwangjin-gu, Seoul 05029, Korea
[2] Faculty of Industrial Design Engineering, Delft University of Technology, Landbergstraat 15, CE 2628 Delft, The Netherlands
* Correspondence: jtpark25@konkuk.ac.kr; Tel.: +82-2-450-3538; Fax: +82-2-450-3504

Supplementary Results

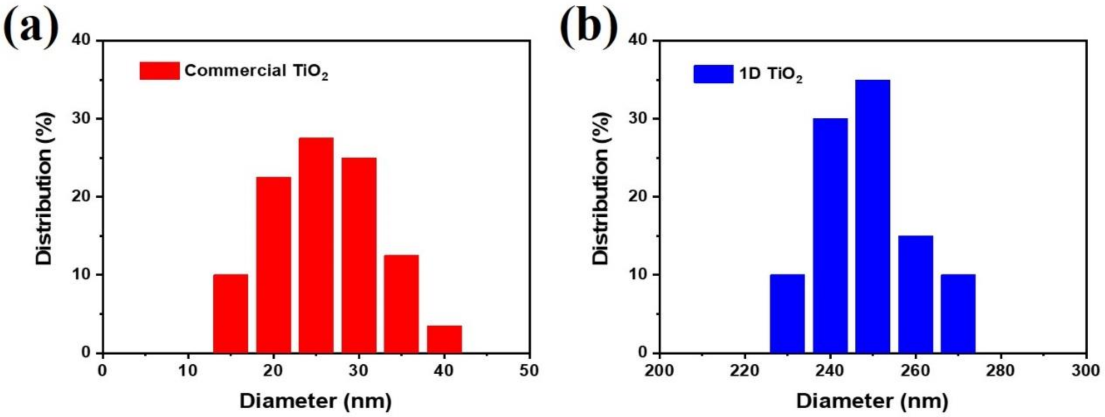

Figure S1. The size distribution of commercial TiO_2 (a) and 1D TiO_2 (b).

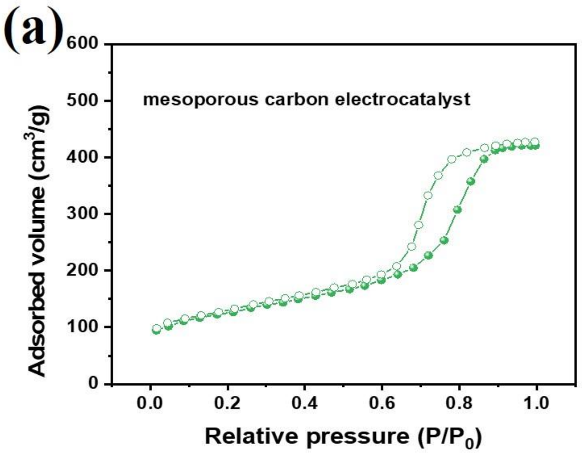

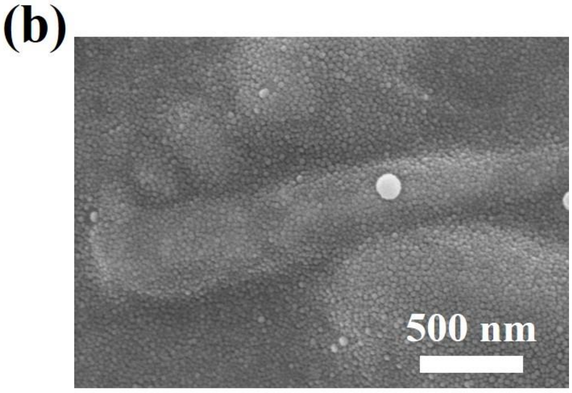

Figure S2. (**a**) N_2 adsorption (filled symbols) and desorption (unfilled symbols) isotherms of mesoporous carbon electrocatalyst derived from PVDC-*g*-POEM double comb copolymer and (**b**) SEM of mesoporous carbon electrocatalyst.

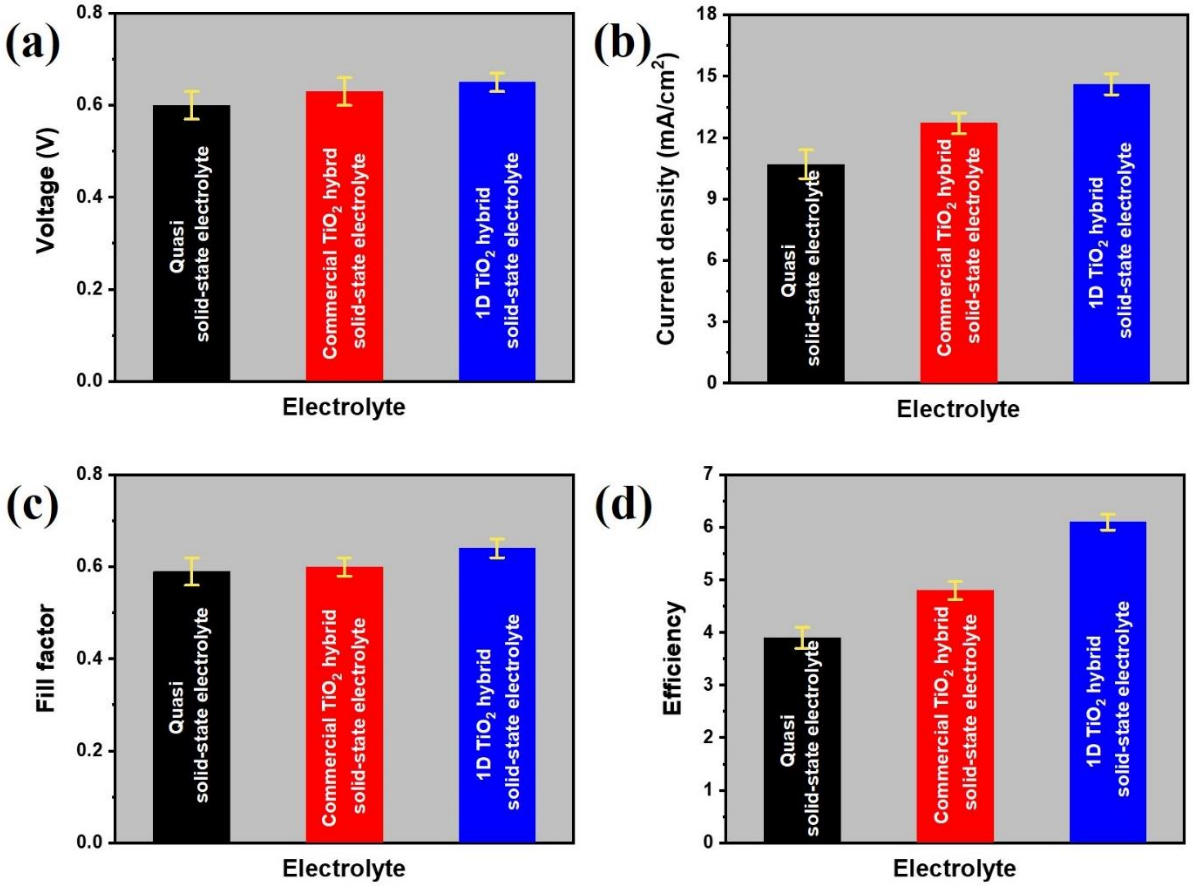

Figure S3. Photovoltaic parameters (a) voltage, (b) current density, (c) fill factor and (d) efficiency of mesoporous carbon based DSSCs fabricated with quasi solid-state electrolyte, commercial TiO_2 hybrid solid-state electrolyte and 1D TiO_2 hybrid solid-state electrolyte at 100 mW/cm^2. Error bars represent the standard deviation of at least 5 cells.

Table S1. Photovoltaic parameters of mesoporous carbon based dye-sensitized solar cells (DSSCs) with quasi solid-state electrolyte, commercial TiO_2 hybrid solid-state electrolyte and 1D TiO_2 hybrid solid-state electrolyte at 100 mW/cm^2 (AM 1.5).

Electrolyte	V_{oc} (V)	J_{sc} (mA/cm^2)	FF	η (%)
Quasi solid-state electrolyte	0.60 ± 0.03	10.7 ± 0.7	0.59 ± 0.03	3.9 ± 0.20
Commercial TiO_2 hybrid solid-state electrolyte	0.63 ± 0.03	12.7±0.5	0.60 ± 0.02	4.8 ± 0.17
1D TiO_2 hybrid solid-state electrolyte	0.65 ± 0.02	14.6±0.5	0.64 ± 0.02	6.1 ± 0.15

Table S2. Comparison of photovoltaic parameters of Pt-free counterelectrode based DSSCs fabricated with solid-state electrolytes reported in the literature.

Counter electrode	Solid-state electrolyte	*Voc* (V)	*Jsc* (mA/cm^2)	*FF*	η (%)	Reference
Meso carbon	1D TiO_2	0.65	14.6	0.64	6.1	This work
PANI	Anionic polymer	0.66	11.8	0.42	4.3	[38]
PEDOT:PSS	PE/S-rGO	0.77	12.5	0.44	4.2	[39]
PAniNT/rGO A	PMMA	0.79	11.5	0.59	5.5	[40]
MoO_3	Polycarbobate	0.66	10.2	0.76	5.1	[41]
Ni0.85Se	PVDF	0.78	13.3	0.70	7.2	[42]
